# Draft Genome Sequence of Endophytic *Herbaspirillum* sp. Strain WT00C, a Tea Plant Growth-Promoting Bacterium

**DOI:** 10.1128/genomeA.01719-16

**Published:** 2017-03-16

**Authors:** Wei Cheng, Guiting Zhan, Weilin Liu, Rong Zhu, Xuejing Yu, Yang Li, Yadong Li, Wenhua Wu, Xingguo Wang

**Affiliations:** Faculty of Life Science, Hubei Collaborative Innovation Center for Green Transformation of Bioresources, Hubei University, Wuhan, Hubei Province, China

## Abstract

Endophytic *Herbaspirillum* sp. strain WT00C was isolated from tea plant (*Camellia sinensis* L.). Here, we report the 6.08 Mb draft genome sequence of this strain, providing bioinformation about its agronomic benefits and capability to reduce selenate/selenite into red elemental selenium.

## GENOME ANNOUNCEMENT

*Herbaspirillum* sp. strain WT00C is an endophytic bacterium isolated from *Camellia sinensis* L. and is classified as a novel member in the genus *Herbaspirillum* (1). It does not exhibit any nitrogen-fixing activity but can produce indole-3-acetic acid (IAA), ammonia, and siderophores (1), similar to endophytic *Herbaspirillum* strains (2–4) and certain plant growth-promoting rhizobacteria (5–7). Inoculating the bacterium into tea plants markedly stimulates plant growth without any disease symptom (8). The strain WT00C enters plants only via plant vulnus, and colonizes specifically in tea plants (8). In addition, this bacterium can effectively reduce selenate/selenite into red elemental selenium. Here, we report the whole-genome draft sequence and annotation of the strain WT00C.

The genome of *Herbaspirillum* sp. strain WT00C was sequenced with an Illumina HiSeq 2000 instrument according to the manufacturer’s instructions. High molecular mass-genomic DNA prepared from the strain WT00C was used to construct small (500 bp) and large (6 kb) random sequencing libraries. In both the 500 bp and 6 kb libraries, the mean read length was 90 bp. The reads were filtered and assembled into contigs using SOAPdenovo v1.05 (http://soap.genomics.org.cn). The resulting sequences were assembled into four scaffolds consisting of 11 contigs based on all the paired-end information of reads. Repeat sequences and gene islands (Gis) were predicted using Tandem Repeat Finder (TRF) (9), IslandPath-DIOMB, and SIGI-HMM software (10). Potential protein-coding regions were identified by an integrated automatic annotation platform with Glimmer 3.0 (11) and BLAST softwares (12). Probable functions of translation products of open reading frames (ORFs) were inferred using the BLAST package to search the public databases nonredundant (nr) NCBI, UniProtKB/Swiss-Port (EMBL-EBI), Inter Pro (13), GO (Gene Ontology) (14), and Kyoto Encyclopedia of Genes and Genomes (KEGG) (15). The draft genome of the strain WT00C consists of 6,079,821 bp with a G+C content of 62.36% and 5,537 ORFs. Protein-coding regions cover 87.1% of the genome sequence. The predicted draft genome contains 57 tRNA genes and nine rRNA genes coding 5S, 16S, and 23S rRNA. The total tandem repeat length is 21,508 bp with 263 tandem repeats, which covers 0.354% genomic DNA. 140 minisatellite and 61 microsatellite DNAs were also predicted, and no plasmid or prophage sequence was found.

The genomic annotation reveales that the strain WT00C holds an ACC deaminase gene and genes related to pathways of indole-3-accetic acid (IAA), siderophore, urea biosynthesis, and selenocompound metabolism, but lacks nitrogen-fixation genes and glycohydrolase genes involved in plant cell wall degradation. This strain also contains genes encoding an intact type IV secretion system (T4SS), which is deficient in other *Herbaspirillum* strains. The genomic information suggests that tea plant-growth promotion by the strain WT00C is achieved by direct and indirect interactions between the bacterium and its host plant.

### Accession number(s).

The genome sequence has been deposited at DDBJ/EMBL/GenBank under the accession number MIJG00000000. The version described in this paper is the first version.

## References

[1] Wang T, Yang S, Chen YX, Hu LL, Tu Q, Zhang L, Liu XQ, Wang XG 2014 Microbiological properties of two endophytic bacteria isolated from tea (*Camellia sinensis* L.) (in Chinese). Acta Microbiol Sin 54:424–432. 25007655

[2] Valverde A, Velázquez E, Gutiérrez C, Cervantes E, Ventosa A, Igual JM 2003 *Herbaspirillum lusitanum* sp. nov., a novel nitrogen-fixing bacterium associated with root nodules of *Phaseolus vulgaris*. Int J Syst Evol Microbiol 53:1979–1983. doi:10.1099/ijs.0.02677-0. 14657133

[3] Baldani JI, Baldani VLD, Seldin L, Dobereiner J 1986 Characterization of *Herbaspirillum seropedicae* gen. nov., sp. nov., a root-associated nitrogen-fixing bacterium. Int J Syst Bacteriol 36:86–93. doi:10.1099/00207713-36-1-86.

[4] Kirchhof G, Eckert B, Stoffels M, Baldani JI, Reis VM, Hartmann A 2001 *Herbaspirillum frisingense* sp. nov., a new nitrogen-fixing bacterial species that occurs in C4-fibre plants. Int J Syst Evol Microbiol 51:157–168. doi:10.1099/00207713-51-1-157. 11211253

[5] Karnwal A 2009 Production of indol acetic acid by fluorescent pseudomonas in the prescence of l-tryptophan and rice root exudates. J Plant Pathol 91:61–63.

[6] Roy M, Basu PS 1992 Studies on root nodules of leguminous plants bioproduction of indole acetic acid by a rhizobium sp. from a twiner *Clitoria ternatea* L. Acta Biotechnol 12:453–460.

[7] Saharan B 2011 Plant growth promoting rhizobacteria: A critical review. Life Sci Med Res 2011:LSMR-21.

[8] Guiting Z, Wei C, Weilin L, Yadong L, Kunming D, Huifu R, Wenhua W, Xingguo W 2016 Infection, colonization and growth-promoting effects of tea plant (*Camellia sinensis* L.) by the endophytic bacterium *Herbaspirillum* sp. WT00C. Afr J Agric Res 11:130–138. doi:10.5897/AJAR2015.10379.

[9] Benson G 1999 Tandem repeats finder: a program to analyze DNA sequences. Nucleic Acids Res 27:573–580. doi:10.1093/nar/27.2.573. 9862982 PMC148217

[10] Langille MG, Hsiao WW, Brinkman FS 2010 Detecting genomic islands using bioinformatics approaches. Nat Rev Microbiol 8:373–382. doi:10.1038/nrmicro2350. 20395967

[11] Delcher AL, Bratke KA, Powers EC, Salzberg SL 2007 Identifying bacterial genes and endosymbiont DNA with glimmer. Bioinformatics 23:673–679. doi:10.1093/bioinformatics/btm009. 17237039 PMC2387122

[12] Altschul SF, Madden TL, Schäffer AA, Zhang JH, Zhang Z, Miller W, Lipman DJ 1997 Gapped BLAST and PSI-BLAST: a new generation of protein database search programs. Nucleic Acids Res 25:3389–3402. doi:10.1093/nar/25.17.3389. 9254694 PMC146917

[13] Mitchell A, Chang HY, Daugherty L, Fraser M, Hunter S, Lopez R, McAnulla C, McMenamin C, Nuka G, Pesseat S, Sangrador-Vegas A, Scheremetjew M, Rato C, Yong SY, Bateman A, Punta M, Attwood TK, Sigrist CJ, Redaschi N, Rivoire C, Xenarios I, Kahn D, Guyot D, Bork P, Letunic I, Gough J, Oates M, Haft D, Huang H, Natale DA, Wu CH, Orengo C, Sillitoe I, Mi H, Thomas PD, Finn RD 2015 The InterPro protein families database: the classification resource after 15 years. Nucleic Acids Res 43:D213–D221. 25428371 10.1093/nar/gku1243 PMC4383996

[14] Tatusov RL, Fedorova ND, Jackson JD, Jacobs AR, Kiryutin B, Koonin EV, Krylov DM, Mazumder R, Mekhedov SL, Nikolskaya AN, Rao BS, Smirnov S, Sverdlov AV, Vasudevan S, Wolf YI, Yin JJ, Natale DA 2003 The COG database: an updated version includes eukaryotes. BMC Bioinformatics 4:41. doi:10.1186/1471-2105-4-41. 12969510 PMC222959

[15] Kanehisa M, Goto S, Hattori M, Aoki-Kinoshita KF, Itoh M, Kawashima S, Katayama T, Araki M, Hirakawa M 2006 From genomics to chemical genomics: new developments in KEGG. Nucleic Acids Res 34:D354–D357. doi:10.1093/nar/gkj102. 16381885 PMC1347464

